# A clock network for geodesy and fundamental science

**DOI:** 10.1038/ncomms12443

**Published:** 2016-08-09

**Authors:** C. Lisdat, G. Grosche, N. Quintin, C. Shi, S.M.F. Raupach, C. Grebing, D. Nicolodi, F. Stefani, A. Al-Masoudi, S. Dörscher, S. Häfner, J.-L. Robyr, N. Chiodo, S. Bilicki, E. Bookjans, A. Koczwara, S. Koke, A. Kuhl, F. Wiotte, F. Meynadier, E. Camisard, M. Abgrall, M. Lours, T. Legero, H. Schnatz, U. Sterr, H. Denker, C. Chardonnet, Y. Le Coq, G. Santarelli, A. Amy-Klein, R. Le Targat, J. Lodewyck, O Lopez, P.-E. Pottie

**Affiliations:** 1 Physikalisch-Technische Bundesanstalt, Bundesallee 100, 38116 Braunschweig, Germany; 2 Laboratoire de Physique des Lasers, Université Paris 13, Sorbonne Paris Cité, CNRS, 99 Avenue Jean-Baptiste Clément, 93430 Villetaneuse, France; 3 LNE-SYRTE, Observatoire de Paris, PSL Research University, CNRS, Sorbonne Universités, UPMC University of Paris 06, 61 Avenue de l′Observatoire, 75014 Paris, France; 4 Réseau National de télécommunications pour la Technologie, l′Enseignement et la Recherche, 23–25 Rue Daviel, 75013 Paris, France; 5 Institut für Erdmessung, Leibniz Universität Hannover, Schneiderberg 50, 30167 Hannover, Germany; 6 Laboratoire Photonique, Numérique et Nanosciences, UMR 5298 Institut d'Optique Graduate School, CNRS, and Université de Bordeaux, 1 Rue F. Mitterrand, 33400 Talence, France

## Abstract

Leveraging the unrivalled performance of optical clocks as key tools for geo-science, for astronomy and for fundamental physics beyond the standard model requires comparing the frequency of distant optical clocks faithfully. Here, we report on the comparison and agreement of two strontium optical clocks at an uncertainty of 5 × 10^−17^ via a newly established phase-coherent frequency link connecting Paris and Braunschweig using 1,415 km of telecom fibre. The remote comparison is limited only by the instability and uncertainty of the strontium lattice clocks themselves, with negligible contributions from the optical frequency transfer. A fractional precision of 3 × 10^−17^ is reached after only 1,000 s averaging time, which is already 10 times better and more than four orders of magnitude faster than any previous long-distance clock comparison. The capability of performing high resolution international clock comparisons paves the way for a redefinition of the unit of time and an all-optical dissemination of the SI-second.

## Abstract (web summary)

Comparing the frequency of two distant optical clocks will enable sensitive tests of fundamental physics. Here, the authors compare two strontium optical-lattice clocks 690 kilometres apart to a degree of accuracy that is limited only by the uncertainty of the individual clocks themselves.

## Graphical abstract

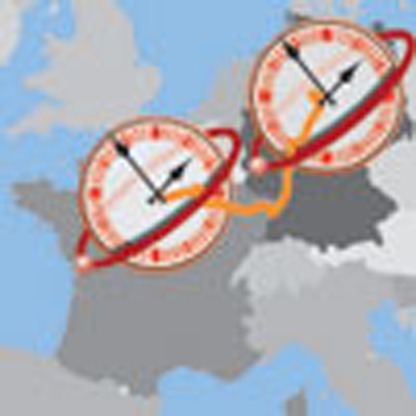

Time and frequency are the most precisely measured physical quantities thanks to atomic clocks. Optical clocks are—due to their orders of magnitude better precision^1^ and accuracy^2^,^3^,^4^ than their microwave counterparts realizing the definition of the SI-second—new sensors with highest resolving power to, for example, test the temporal stability of fundamental constants^5^,^6^,^7^,^8^. Complemented by phase-coherent telecom fibre links^9^,^10^,^11^,^12^ as extremely powerful tools for frequency dissemination on a continental scale, new scenarios for fundamental and applied science will become realizable through large-scale fibre-based optical clock networks. A prominent example is the search for dark matter^13^ by monitoring the local time scale jitter when the Earth moves through cosmic domains. Accurate mapping of the relative gravitational shift of clocks will enhance redshift tests^14^ performed with clocks in space (Atomic Clock Ensemble in Space (ACES)). Highly accurate clock networks can also perform tests of the Einstein equivalence principle^15^ by searching for frequency modulation between clocks in a network moving in the Sun's gravitational potential, or can provide a better synchronization between very long baseline interferometers. Additionally, new geodetic reference frames based on relativistic geodesy^16^,^17^,^18^ or a quantum network of clocks^19^ can be established.

The traditional means to compare remote optical clocks are using microwave signals: the optical clocks' frequencies are measured against primary caesium microwave clocks and the frequency values measured locally are compared, or satellite-based methods transferring the frequency are applied^20^. The accuracy of the former method is limited by the caesium microwave clocks with the most stringent bound set by our groups^5^,^21^,^22^,^23^ at an agreement within 4 × 10^−16^; satellite link techniques do not reach significantly better performance either. Hence, the capabilities of distant optical clocks could so far neither be fully tested nor exploited.

Here, we overcome these limitations by a direct, all-optical frequency comparison between two optical clocks via a telecom fibre link and demonstrate a remote optical clock comparison with one order of magnitude better accuracy and orders of magnitude shorter averaging time than with microwave clocks or satellite links. Our work not only enables the above mentioned applications, but also clears the path towards a redefinition of the unit of time, the SI second^24^,^25^ through regular and practical international comparisons of optical clocks. They are of utmost importance for an evolution of the international time scale temps atomique international (TAI), which delivers the accuracy of primary frequency standards to a wide range of users in science and society.

## Results

### Experimental layout

**Figure 1:**
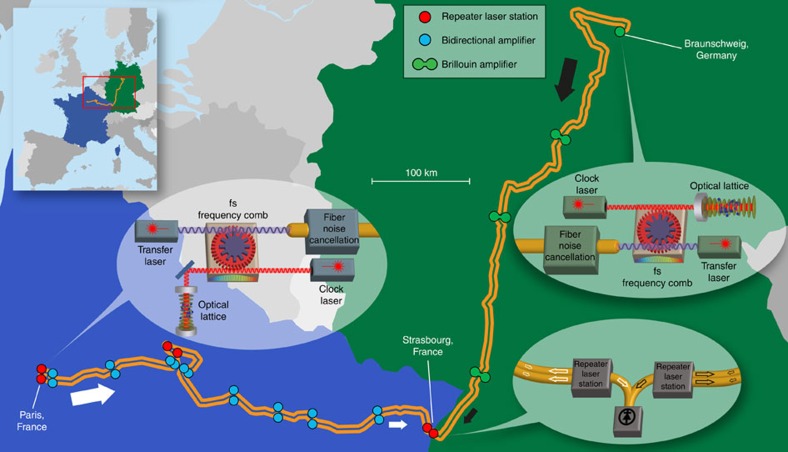
Schematic of the clock comparison. The strontium lattice clocks are located at the national metrology institutes SYRTE and PTB in Paris and Braunschweig, respectively. The course and lay-out of the fibre link sections to Strasbourg (705 km from Paris, 710 km from Braunschweig) are indicated on the map. Additionally, the individual setups consisting of a clock laser, optical lattice, fs frequency comb, transfer laser and stabilized link are shown schematically. In Strasbourg, the frequency difference between the transfer lasers is measured. For details see the main text and Methods. Country border data available at http://thematicmapping.org/ under a Creative Commons Attribution-ShareAlike 3.0 Unported. Full terms at http://creativecommons.org/licenses/by-sa/3.0/.

Our experimental set-up is composed of two optical clocks and an optical frequency transfer system to allow their direct optical comparison. The two strontium optical lattice clocks Sr_SYRTE_ and Sr_PTB_ are located at the national metrology institutes LNE-SYRTE in Paris^5^,^23^, France and PTB in Braunschweig^21^,^22^, Germany. At each institute, femtosecond (fs) frequency combs are used to accurately measure the frequency ratio of the optical clock to a transfer laser. Two stabilized fibre links transfer, respectively, the frequency information of the lattice clocks from Paris^26^ and Braunschweig^27^ to the connection point in Strasbourg (Fig. 1).

### Optical clock performance

The Sr lattice clocks provide a reproducible and long-term stable optical frequency at 429 THz (698 nm) by locking the frequency of an ultra-stable laser^5^,^23^,^28^ with sub-Hertz line width to the ultra-narrow atomic ^1^S_0_–^3^P_0_ transition of laser-cooled ^87^Sr atoms. The atoms are trapped in an optical lattice near the magic wavelength^29^ (see Methods) and are thus practically immune to motional effects such as Doppler shifts.

Both lattice clocks interrogate a few hundred atoms trapped inside a vacuum chamber in an optical lattice that is operated at or close to the Stark shift cancellation wavelength at 813 nm of the Sr clock transition ^1^S_0_–^3^P_0_ at 698 nm or 429 THz^5^,^21^. The lattice orientation is vertical in Sr_SYRTE_, while a nearly horizontal lattice is used in Sr_PTB_. To load the atoms into the lattice, Zeeman slowing of a Sr atomic beam and laser cooling in a magneto-optical trap (MOT) operated on the ^1^S_0_–^1^P_1_ transition are performed.

The atoms in the MOT are either loaded into a deep lattice, which is compatible with the millikelvin temperature of the atoms, and cooled in the lattice on the intercombination line ^1^S_0_–^3^P_1_ (Sr_SYRTE_); or they are first transferred to a second-stage MOT operating on the same transition for further cooling and then loaded in a shallow lattice (Sr_PTB_). The atoms are alternatingly state-prepared in the stretched Zeeman levels |*m*_*F*_|=9/2, from which Δ*m*_*F*_=0 transitions are driven by Rabi interrogation with a pulse length of 580 ms in a small magnetic bias field (Sr_PTB_) or a pulse length of 200 ms in a larger magnetic bias field of 1.6 × 10^−4^ T (Sr_SYRTE_).

Physical effects leading to offsets of the laser frequency from the unperturbed atomic frequency are quantified in the uncertainty budgets for both optical clocks (Table 1 and Methods). The Sr ^1^S_0_–^3^P_0_ transition frequency is expected to be realized in Paris and Braunschweig with respective fractional uncertainties of 4 × 10^−17^ and 2 × 10^−17^ and instabilities of about 5 × 10^−16^ (*τ*/s)^−1/2^ to 3 × 10^−15^ (*τ*/s)^−1/2^ for averaging times *τ*>10 s. The details of the actual implementation of state preparation and interrogation being quite different in the two experimental realizations of a Sr lattice clock^5^,^21^,^22^,^23^, systematic and statistical uncertainties are highly independent and uncorrelated. Our comparisons thus provide very stringent tests of the clocks' performance.

### Optical link between the clocks

The two Sr lattice clocks are separated by 690 km in line-of-sight and by 1,415 km of actual fibre links. Measuring the frequency ratio of the two lattice clocks involves optical frequency combs^30^,^31^ at each institute to compare the local clock laser's frequency with a narrow linewidth transfer laser^32^ in the telecommunication band at 194.4 THz (1,542 nm). By counting the radio frequency beat-note signals of the lasers with the frequency combs, the optical frequency ratios between each Sr clock and the associated transfer laser are determined accurately with less than 10^−18^ uncertainty^5^,^21^. The transfer lasers are injected from Paris and Braunschweig into two long-haul coherent fibre links, both ending at the University of Strasbourg. There, the beat note between the two transfer lasers is recorded, thus enabling an uninterrupted frequency transfer between the institutes.

The main challenge is to ensure that the coherent phase of the transfer laser is preserved over the total distance of the link, despite the phase noise imprinted on the light by environmentally induced optical path length fluctuations of the fibre^9^,^11^,^26^,^27^,^33^. For this purpose, part of the light reaching the remote end of the link is sent back through the same fibre; thus the round-trip phase noise is detected at the sender's position and actively cancelled, and we accurately transfer the frequency at the input of the fibre to the remote end^34^,^35^,^36^ (see Methods).

The power in each optical fibre path is attenuated by about 20 orders of magnitude (205 dB in France, 178 dB in Germany); the attenuation is mostly compensated by bidirectional amplifiers (see Methods). The experiment combines two approaches: in France, repeater laser stations (RLSs) and broad-band amplifiers transfer an optical frequency in parallel with internet traffic^26^, while in Germany we use a small number of narrow-band, high-gain amplifiers^27^ on a dedicated fibre. Both links are established as a cascaded up- and down-link in a loop-back configuration to and from Strasbourg, to allow assessment of the accuracy of the frequency transfer. This is achieved using RLSs^10^,^26^ in Strasbourg, which also compare the signals from the two links at Strasbourg (see Fig. 1).

**Figure 2:**
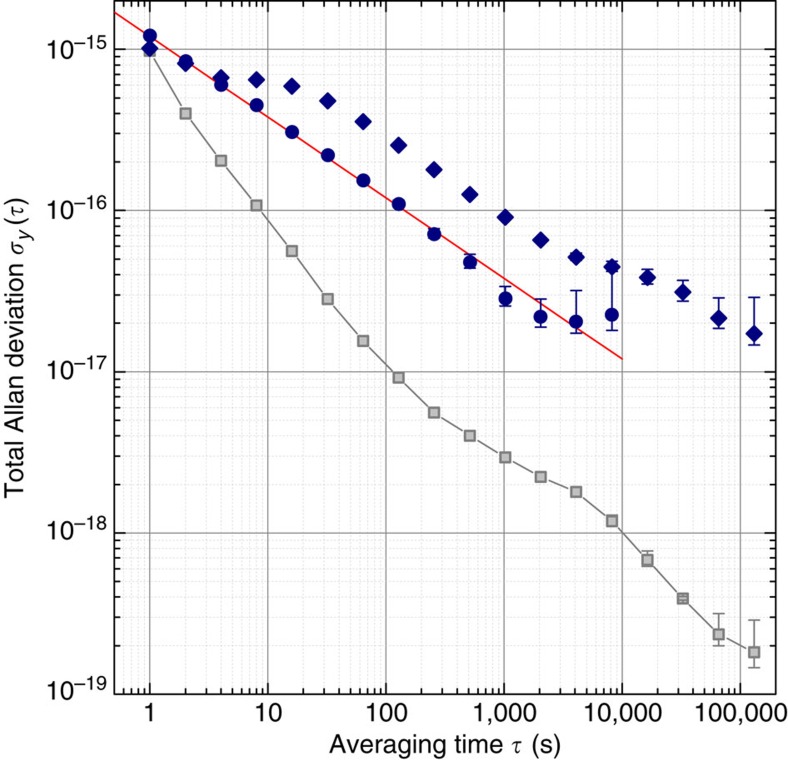
Instability of the clock comparison. The instability is expressed as total Allan deviation *σ*_*y*_, as a function of averaging time *τ* for the first (circles; modified Julian date MJD 57092) and second measurement campaign (diamonds; MJD 57177–57198). We attribute the slightly different instabilities to different clock performances. An instability of 1.2 × 10^−15^ (*τ*/s)^−1/2^ is observed during the first campaign (red line). A statistical measurement uncertainty of 2 × 10^−17^ is thus achieved after only 3,000 s measurement time. The squares with connecting lines show the link's contribution to the statistical uncertainty for the data recorded in the 2nd campaign (see Methods). The error bars indicate 1σ uncertainties.

The relative frequency instabilities of the French and German links are 8 × 10^−16^ and 1 × 10^−15^, respectively, at 1 s integration time. They average down faster than the clocks (Fig. 2) and thus contribute negligibly to the instability of the measurement. The fractional uncertainty of the whole link^26^,^27^ is assessed to be 2.5 × 10^−19^ (see Methods). Such long-distance links with uncertainties on the order of 10^−19^ open the route for comparisons of even more accurate clocks and novel high precision experiments.

### Campaigns to compare the strontium optical clocks

The frequency ratio 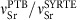 is determined accurately by combining three frequency ratio measurements from Paris, Strasbourg and Braunschweig.

Approximating the frequency ratios involving the strontium and transfer laser frequencies (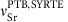 and 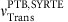) by the difference of the respective fractional frequencies *y*(*t*)=[*ν*(*t*)−*ν*_0_]·*ν*_0_^–1^ with the frequency *ν*(*t*) measured at the time *t* and the nominal frequency *ν*_0_, the clock ratio reads as:


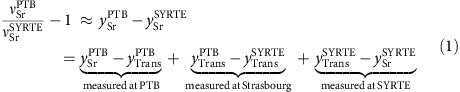


The combination of such measurements requires an accurate synchronization of the counters to avoid an additional frequency offset and instability contribution^9^,^37^ (see Methods). In this set-up we performed two sessions of measurements in March and June 2015. We present here 145 h of accumulated data. The frequency ratio is corrected for offsets due to systematic frequency shifts and biases due to synchronization offsets of the counters (Table 1 and Methods).

A key aspect among the corrections for remote clock comparisons at the 10^−17^ level and beyond is the correction for the differential relativistic redshift effect. This requires a state-of-the-art determination of the gravity potential at both clock sites, where, besides the global long-wavelength and eventually temporal gravity field variations, especially the local spatial influence of the Earth's gravity potential (see Methods) on the clock frequency needs to be considered. The determination of the gravity potential involved a combination of global positioning system (GPS) based height measurements, spirit levelling and a geoid model refined by local gravity measurements. For our clock comparisons, the applied average correction is (−247.4±0.4) × 10^−17^. The differential temporal variation of the gravity potential by tides will become relevant in the mid-10^−18^ range of uncertainty for clocks separated as in the experiment here. The absolute variation of the gravity potential can, however, produce fractional frequency changes relative to an ideal reference of few 10^−17^.

The instability of the fractional frequency ratio observed between Sr_SYRTE_ and Sr_PTB_ is represented by the total Allan deviation in Fig. 2. A long-term averaging behaviour consistent with a white frequency noise level of about 1 × 10^−15^ (*τ*/s)^−1/2^ for the first run and 3 × 10^−15^ (*τ*/s)^−1/2^ for the second one is observed. This confirms that the link instability is negligible for these times. After less than an hour of averaging, we have reached a statistical uncertainty of 2 × 10^−17^ in the first campaign. We emphasize that this level of precision is unprecedented for remote clock measurements and not achievable by any other present-day means of remote frequency comparison.

**Figure 3:**
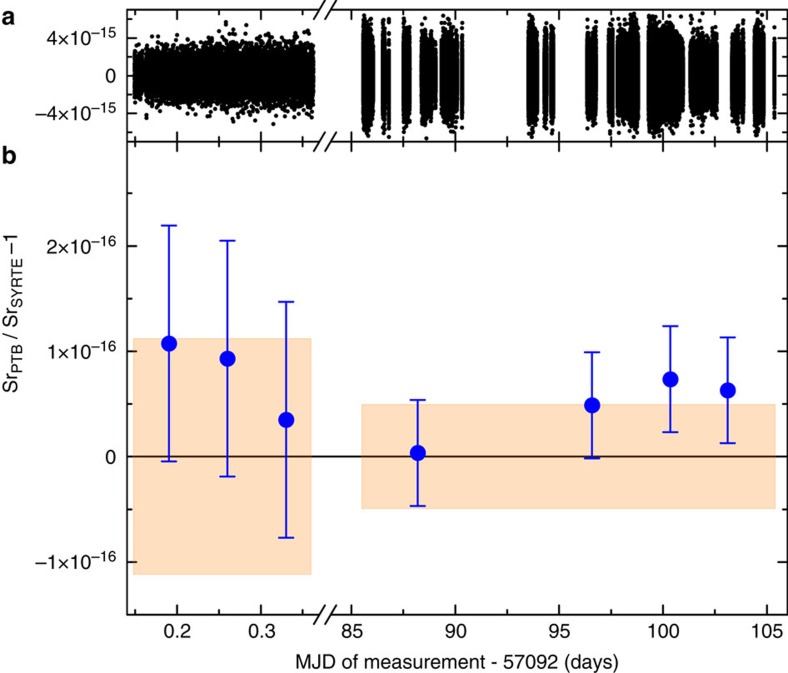
Frequency ratio (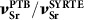
−1). (**a**) Time trace of the valid data. (**b**) Aggregate segments of 6,000 s length (1st campaign) and 125,000 s (2nd campaign). Error bars (1σ) include statistical and systematic uncertainties (Table 1). The error bars are dominated by the systematic uncertainty, which is not reduced further by averaging. On average, the clocks agree within the ±1σ interval of uncertainty (shaded area) around the expected result of zero, demonstrating the very good agreement between the two systems.

This international all-optical clock measurement surpasses the accuracy of the best possible comparison achievable by measurements against primary caesium clocks by one order of magnitude^5^,^21^,^22^,^23^. In Fig. 3, we show the averaged deviation of the frequency ratio 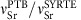 from unity for three 6,000 s long segments of the first campaign and for four segments of the second campaign covering 125,000 s each. Their respective lengths are chosen to be close to the maximum averaging time for which we can determine the statistical measurement uncertainty (Fig. 2). For both measurement sessions we find agreement between the two fully independent and remote optical clocks within the combined uncertainties. For the more extensive second campaign, the fractional offset between the two clocks is (4.7±5.0) × 10^−17^. Showing optical clock agreement over long distances is an important step towards a redefinition of the SI-second. In addition, the measurement time required for a comparable accuracy is reduced by four orders of magnitude compared with measurements with primary clocks. This allows investigating time-dependent (for example, diurnally varying) clock data, opening up completely new research capabilities^5^,^13^,^15^.

## Discussion

Comparing optical clocks is essential for their development and evaluation. For building confidence in them, using clocks of the same type is especially appealing, since the true ratio is known to be unity. The clocks have to fulfil this requirement within their combined uncertainty. For state-of-the-art optical clocks it is especially difficult to obtain highest quality results since clocks with similarly high performance are required. Still, excellent comparisons have been performed for, Al^+^ ion clocks^4^ on the level of 2.5 × 10^−17^, for example. An uncertainty level of 5 × 10^−17^ has been achieved involving the Sr clock with the smallest individual uncertainty^38^, surpassed only by one other Sr–Sr comparison^3^ with an uncertainty of 5 × 10^−18^. However, all experiments on this low level of uncertainty have been performed within a single group, often with very similar devices that exhibit common mode uncertainty contributions^3^. In contrast, the experiments we have presented here involve two fully independent apparatuses and teams, which adds to the significance of the 5 × 10^−17^ clock agreement.

Beyond the results presented here, and taking advantage of an ongoing refinement of fibre links and optical clocks, the addition of further institutes will establish a continental, clock science fibre network. Even for greatly improved optical clocks, running continuously with an instability below 10^−16^ (*τ*/s)^−1/2^, our fibre link instability still does not pose a limitation due to its faster averaging behaviour. Such long-distance clock comparisons will soon have reached sufficiently high accuracy that the uncertainty of the geo-potential of mid-10^−18^ will be within the measurement range of optical frequency standards. This will turn them into precise and accurate height monitors at the centimetre level over continental distances and paves the path towards a new height reference frame based on clocks.

## Methods

### Optical lattice clock operation details

Light to drive the 698 nm transition is provided by diode-laser systems that are frequency-stabilized to high-finesse optical resonators^28^. Each Zeeman transition in sequence is interrogated approximately at both half-width points. Through comparison of the detected average excitation probabilities of the atomic sample on both sides of the line, an error signal is derived to steer the frequency of the interrogation laser to the atomic transition frequency. Interrogation of the *m*_*F*_=±9/2 levels effectively cancels the linear Zeeman shift and measures the magnetic field at the same time. The cooling, preparation and interrogation sequence requires about 1 s.

Light from the interrogation laser is also sent to a fs frequency comb that phase-coherently bridges the frequency gap between the 429 THz clock transition and the transfer lasers at 194.4 THz transmitted through the fibre links.

### Optical lattice clock uncertainty evaluation

The residual lattice light shift is evaluated by running the clock successively with different trap depths spanning from 50 *E*_R_ to 500 *E*_R_ (SYRTE) or 70 *E*_R_ to 150 *E*_R_ (PTB), where *E*_R_ is the lattice recoil energy. Higher-order light shifts^39^ are accounted for by a non-linear regression (SYRTE) or a correction of the data (PTB). The density shift is obtained by interleaving high density (about five atoms per site) and low density (about two atoms per site) sequences. No frequency shift is observed between these sequences within the statistical resolution. The quadratic Zeeman shift is evaluated by fitting the clock frequency as a function of the magnetic field obtained from the splitting between the two Zeeman components. The probe light shift is evaluated by a theoretical calculation of the AC polarizability of the Sr clock levels. The AOM phase chirp is both measured by interferometric measurements and by measuring the shift induced in the clock frequency. Line pulling is evaluated by running the clock with different linewidths (SYRTE) or by evaluating possible spurious excitation amplitudes (PTB). To evaluate the black-body radiation shift, calibrated Pt100 temperature sensors are placed around the vacuum chamber spanning the coldest and hottest points. The temperature spreads are 0.9 K (SYRTE) and 0.6 K (PTB), corresponding to uncertainties of 0.26 K and 0.17 K.

### Link noise cancellation

Phase-coherent frequency transmission through the fibre link is achieved by active cancellation^34^,^35^,^36^ of the propagation noise that originates mainly from environmentally induced fluctuations of the fibre's index of refraction. The round-trip fibre noise is measured and corrected at one end. When the light goes forth and back through the same fibre, as in our set-up, the corresponding static fibre noise contributions are almost equal and can be rejected by more than five orders of magnitude, even for long-haul fibre links^9^,^26^,^27^. We achieve high signal-to-noise detection by using heterodyne detection techniques that allows us to remove the influence of parasitic reflections and scattering^34^,^36^.

The 705 km-long optical link from SYRTE to Strasbourg uses fibres of the French academic network with parallel data traffic. RLSs^10^,^26^ repeat the optical phase of an ultra-stable laser operated at 194.4 THz (1,542 nm) and build a cascaded link of two spans from Paris to Reims and Reims to Strasbourg. Each RLS contains a laser that is phase-locked to the incoming signal and sent both to the previous and the next link for phase-noise compensation. To ensure continuous and bidirectional propagation along the fibres, the telecommunication nodes and the amplifiers are bypassed. In parallel, a similar down-link from Strasbourg to Paris is established to check the integrity of the frequency dissemination. The 710 km-long optical link from Braunschweig to Strasbourg uses fibre Brillouin amplification^27^ and an RLS at Strasbourg to cascade this up-link with a parallel down-link from Strasbourg to Braunschweig for evaluation purposes.

At Strasbourg, both links are terminated by a dedicated RLS. Their local lasers are phase-locked to the respective frequency of the transfer lasers arriving from SYRTE and PTB. The difference frequency of the RLSs' local lasers is counted with respect to a low-noise ultra-stable radio frequency oscillator disciplined to a GPS signal. The GPS receiver also delivers a pulse-per-second signal by which the counters in Strasbourg are synchronized. The counters in Paris and Braunschweig are synchronized to pulse-per-second signals derived from UTC(OP) and UTC(PTB).

### Instability and uncertainty contribution by the link

To characterize the link uncertainty contribution to the clock comparison, the links were operated using an up- and down-link, in a loop configuration. The proper operation of the link is validated analysing the frequency offset between the light injected to the up-link with respect to the light received back from Strasbourg on the down-link. This gives an upper limit for the instability and uncertainty of the frequency delivered to Strasbourg.

German link data were selected for the valid time intervals of the clock comparison and thus contain interruptions (Fig. 3). For this data set, we calculated the total Allan deviation: this represents the statistical measurement uncertainty contribution of this part of the link. For the French link, we deduce a slightly smaller statistical uncertainty from shorter, continuous recordings and from the detailed discussion in ref. 26. The short connection between the two RLSs in Strasbourg contributes negligibly to the overall link uncertainty and instability. Therefore, the total statistical uncertainty is well represented by the data obtained on the German section, and its total Allan deviation that is shown in Fig. 2.

The systematic uncertainty of the German link during the second campaign is <1.5 × 10^−19^, that is, there is no offset within the statistical uncertainty shown in Fig. 2. The French link has been characterized to show a systematic uncertainty <2 × 10^−19^. The relative frequency difference between both links is measured at Strasbourg with an uncertainty of 2 × 10^−20^. The overall link systematic uncertainty is calculated as the quadratic sum of these three contributions, yielding a value of <2.5 × 10^−19^.

### Correction for gravity potential difference

Clocks experience a fractional frequency shift that is proportional to *W·c*^−2^ with the gravity potential *W* (including a gravitational and a centrifugal component) and the speed of light *c*. The gravity potential difference between the position of the atoms in each lattice clock and a nearby reference marker was found from spirit levelling and the measured local gravity acceleration of the Earth, while the gravity potential of the reference markers was determined by GPS measurements of the height with respect to a rotational ellipsoid in combination with a geoid model refined by local gravity measurements^40^. In this way we are able to correct the redshift between both clocks with an uncertainty equivalent to about 4 cm in height, considering the accuracy of the geoid model as well as the GPS and levelling measurements.

### Counter synchronization and correlation analysis

During the first measurement campaign, the gate intervals of the remotely operated frequency counters in Strasbourg and Paris were accidentally not synchronized with local realizations of the Coordinated Universal Time (UTC). Via the fibre link, only the synchronization between the counters in Braunschweig and Strasbourg could be measured accurately, using the methods described in ref. 37. Therefore, the synchronization between the counters in Paris and in Strasbourg was derived indirectly.

In case of a frequency drift 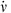 of a transfer laser and a time offset of the counter gates Δ*T*, a frequency offset 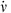
·Δ*T* will be found^9^,^37^. The infrared lasers used for frequency transfer exhibited a drift of up to 2 Hz s^−1^. Furthermore, the lasers' noise will not be fully cancelled from the measurement since the measurements are not fully correlated. This can be used to retrieve Δ*T* from the data^41^ by determining the amount of noise as represented by the Allan deviation *σ*_*y*_(*τ*=1 s, 2 s) for counter data sets that are interpolated on a dense temporal grid and are shifted with respect to each other. The Allan deviations *σ*_*y*_ show a pronounced minimum for the actual Δ*T* as was tested for the data measured in Braunschweig and Strasbourg. In this way we could retrieve the missing counter synchronization between Paris and Strasbourg with an uncertainty of 10 ms. The data analysis was then performed based on the shifted datasets such that no further correction had to be applied. Together with the drift rate 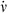 of the transfer laser between Paris and Strasbourg, this uncertainty causes the counter synchronization uncertainty of 10^−16^ in Table 1 for the first measurement.

During the second measurement campaign, the drift rate 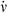 of the link lasers was kept below 50 mHz s^−1^ (that is, 2.5 × 10^−16^ s^−1^ relative), and the counters were synchronized to within Δ*T*=0.2 ms. Hence the uncertainty contribution to the Sr–Sr frequency measurement due to synchronization issues, that is, 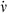
·Δ*T*, is below 20 μHz, or 1 × 10^−19^ in relative terms. No correlation analysis was therefore required and applied to the data.

### Frequency counter data recording

Depending on the dominant noise type, the Allan deviation representing the statistical measurement uncertainty follows characteristic power laws for the temporal averaging of the recorded signals. High suppression of phase noise as present in the fibre link can be achieved by appropriate weighting functions for averaging the frequency data recorded by the dead-time-free counters^27^,^42^. Therefore, we operate all counters in a Λ-averaging mode with 1 s gate time, which effectively represents a low-pass filter. The Allan deviation in Fig. 2 shows an averaging behaviour which is consistent with white frequency noise. Thus, a uniformly (or Π-) weighted averaging of the 1 s-Λ-averaged data leads to a frequency average with a smaller statistical uncertainty that is represented by the Allan deviation at the given averaging time *τ*.

### Data availability

The data that support the findings of this study are available from the corresponding authors on request.

## Additional information

**How to cite this article:** Lisdat, C. *et al.* A clock network for geodesy and fundamental science. *Nat. Commun.* 7:12443 doi: 10.1038/ncomms12443 (2016).

## Figures and Tables

**Table 1:** Uncertainty budget.

Clock uncertainty	Sr lattice clock Paris		Sr lattice clock Braunschweig	
	Corr. (10^−17^)	Unc. (10^−17^)	Corr. (10^−17^)	Unc. (10^−17^)
First and higher-order lattice LS	0	2.5	−1.1	1.0
Black-body radiation	515.5	1.8	492.9	1.3
Black-body radiation oven	0	1.0	0.9	0.9
Density shift	0	0.8	0	0.1
Quadratic Zeeman shift	134.8	1.2	3.6	0.15
Line pulling	0	2.0	0	<<0.1
**Total clocks**	**650.3**	**4.1**	**496.3**	**1.9**

**Ratio Sr**_**PTB**_**/Sr**_**SYRTE**_	**Campaign I Unc. (10**^−**17**^**)**	**Campaign II Unc. (10**^−**17**^**)**
Systematics Sr_SYRTE_	4.1	4.1
Systematics Sr_PTB_	2.1	1.9
Statistical uncertainty	2	2
fs combs	0.1	0.1
Link uncertainty	<0.1	0.03
Counter synchronization*	10	<0.01
Gravity potential correction†	0.4	0.4
**Total clock comparison**	**11.2**	**5.0**

Corr., fractional correction; LS, light shift; Unc., fractional uncertainty.

The numbers vary slightly over the course of the measurement. All uncertainties are 1σ.

^*^Frequency counters have been synchronized in the second campaign.

^†^The applied gravity potential correction is −247.2 × 10^−17^, see text.

Bold entries represent the sum of all the individual contributions listed before rather than another contribution.
